# Aberrant functional connectivity between the retrosplenial cortex and hippocampal subregions in amnestic mild cognitive impairment and Alzheimer’s disease

**DOI:** 10.1093/braincomms/fcae476

**Published:** 2024-12-31

**Authors:** Junkai Wang, Shui Liu, Peipeng Liang, Bin Cui, Zhiqun Wang

**Affiliations:** Department of Radiology, Aerospace Center Hospital, Beijing 100049, China; Department of Radiology, Aerospace Center Hospital, Beijing 100049, China; School of Psychology, Capital Normal University, Beijing 100048, China; Department of Radiology, Aerospace Center Hospital, Beijing 100049, China; Department of Radiology, Aerospace Center Hospital, Beijing 100049, China

**Keywords:** Alzheimer’s disease, amnestic mild cognitive impairment, resting-state functional magnetic resonance imaging, retrosplenial cortex, hippocampal subregions

## Abstract

The posterior cingulate cortex and hippocampus are the core regions involved in episodic memory, and they exhibit functional connectivity changes in the development and progression of Alzheimer’s disease. Previous studies have demonstrated that the posterior cingulate cortex and hippocampus are both cytoarchitectonically heterogeneous regions. Specifically, the retrosplenial cortex, typically subsumed under the posterior cingulate cortex, is an area functionally and anatomically distinct from the posterior cingulate cortex, and the hippocampus is composed of several subregions that participate in multiple cognitive processes. However, little is known about the functional connectivity patterns of the retrosplenial cortex or other parts of the posterior cingulate cortex with hippocampal subregions and their differential vulnerability to Alzheimer’s disease pathology. Demographic data, neuropsychological assessments, and resting-state functional magnetic resonance imaging data were collected from 60 Alzheimer’s disease participants, 60 participants with amnestic mild cognitive impairment, and 60 sex-matched normal controls. The bilateral retrosplenial cortex, other parts of the posterior cingulate cortex, and hippocampus subregions (including the bilateral anterior hippocampus and posterior hippocampus) were selected to investigate functional connectivity alterations in amnestic mild cognitive impairment and Alzheimer’s disease. Resting-state functional connectivity analysis demonstrated heterogeneity in the degree of connectivity between the hippocampus and different parts of the total posterior cingulate cortex, with considerably greater functional connectivity of the retrosplenial cortex with the hippocampus compared with other parts of the posterior cingulate cortex. Furthermore, the bilateral retrosplenial cortex exhibited widespread intrinsic functional connectivity with all anterior–posterior hippocampus subregions. Compared to the normal controls, the amnestic mild cognitive impairment and Alzheimer’s disease groups showed different magnitudes of decreased functional connectivity between the retrosplenial cortex and the contralateral posterior hippocampus. Additionally, diminished functional connectivity between the left retrosplenial cortex and right posterior hippocampus was correlated with clinical disease severity in amnestic mild cognitive impairment subjects, and the combination of multiple functional connectivity indicators of the retrosplenial cortex can discriminate the three groups from each other. These findings confirm and extend previous studies suggesting that the retrosplenial cortex is extensively and functionally connected with hippocampus subregions and that these functional connections are selectively affected in the Alzheimer’s disease continuum, with prominent disruptions in functional connectivity between the retrosplenial cortex and contralateral posterior hippocampus underpinning episodic memory impairment associated with the disease.

## Teaser

Wang *et al*. investigated specific functional connectivity between retrosplenial cortex and hippocampal subregions and to characterize its alterations in Alzheimer’s disease. The authors demonstrated that retrosplenial cortex is extensively connected with hippocampal subregions, with prominent disruptions in connectivity between retrosplenial cortex and contralateral posterior hippocampus in the Alzheimer's disease continuum.

## Graphical Abstract

**Graphical Abstract:**
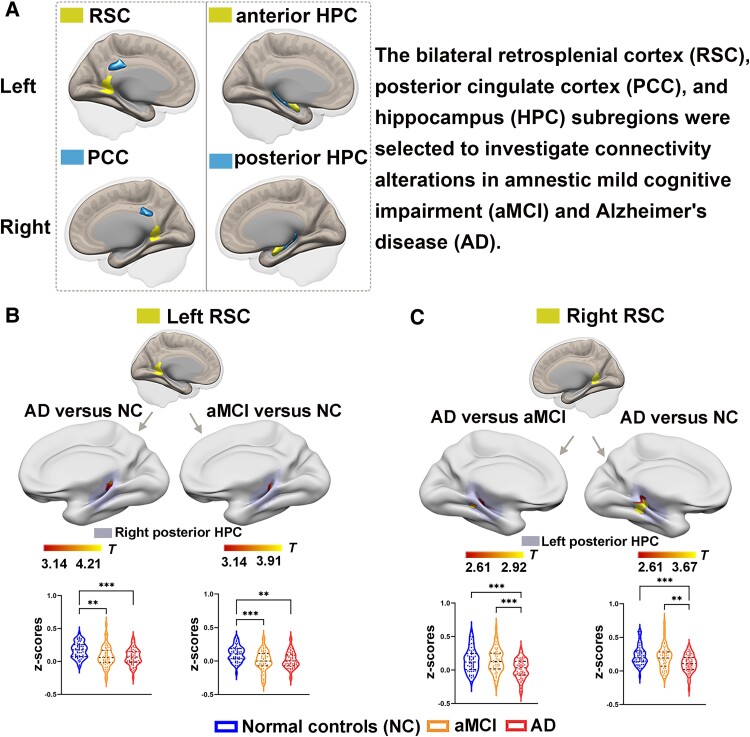

## Introduction

Alzheimer’s disease is a progressive neurodegenerative disorder that is characterized by initial episodic memory impairment and cognitive decline.^1^ Currently, the early diagnosis and prevention of Alzheimer’s disease is a key link to postpone the progression of the disease. Amnestic mild cognitive impairment (aMCI) is considered the most important at-risk state for developing Alzheimer’s disease and represents a probable transitional stage between normal ageing and very early dementia.^2,3^ When considering that episodic memory decline is the earliest and most widely recognized hallmark symptom of Alzheimer’s disease, it is crucial to understand neural alterations underlying memory decline in aMCI and to further explore possible biomarkers for identifying Alzheimer’s disease in its prodromal stage.

Resting-state functional magnetic resonance imaging (rs-fMRI) is a fundamental tool offering a method to detect brain regions that show correlated blood oxygen level-dependent signal fluctuations over time,^4,5^ which reflects intrinsic functional connectivity of brain networks. By using this method, the default mode network (DMN) and the hippocampus (HPC) are known to have essential roles in cognitive processes (especially episodic memory) and have been extensively reported to be affected in aMCI and Alzheimer’s disease.^6-11^ The DMN comprises a network of dynamically coupled brain regions, including the posterior cingulate cortex (PCC)/retrosplenial cortex (RSC), the medial prefrontal cortex (mPFC), and the lateral parietal cortex, that demonstrate greater activity during internal than task-oriented states.^12,13^ Within the DMN subregions, the PCC/RSC is a core region and has been widely considered in dementia research, as it appears to be hypometabolic and is vulnerable to amyloid pathology in the early stages of Alzheimer’s disease, which manifests prior to the onset of clinical symptoms.^14,15^ In previous studies, the RSC has been typically considered a part of the total PCC using the PCC as a region of interest (ROI). However, these studies have demonstrated inconsistent results for MCI participants, and some studies have reported decreased functional connectivity in the total PCC when comparing MCI to healthy older adults, whereas other studies have suggested increased functional connectivity.^16-19^ One possible reason for the discrepant findings is that the PCC is considered an anatomically and functionally heterogeneous region. The RSC is a small area located in the PCC around the splenium^20^; therefore, it is usually functionally misattributed to other posterior cingulate areas.^21^ Human and animal studies have indicated the importance of distinguishing the RSC from other parts of the PCC due to many morphological and connectivity differences.^22,23^ One recent rs-fMRI study also demonstrated that the RSC can be functionally dissociated from other parts of the PCC, thus showing different functional connectivity patterns in prodromal Alzheimer’s disease compared to cognitively normal individuals.^24^ Taken together, the RSC and other parts of the PCC should be differentiated, and they may play separable functional roles during Alzheimer’s disease progression.

In addition to the PCC/RSC, the HPC, which is a structure that is crucial for episodic memory, is of vital importance to the understanding of Alzheimer’s disease progression.^25^ Interactions between the HPC and the DMN nodes, especially the PCC/RSC, have been demonstrated to underlie a range of functions, including episodic memory in old individuals.^26-28^ However, some studies have reported inconsistent results.^29,30^ The discrepant findings could also be attributed to the heterogeneous structural and functional architecture of the PCC. Animal research has found that the density of RSC-HPC connections is much higher than the density of HPC connections to adjacent PCC areas and that the RSC (in conjunction with the HPC) is important to episodic memory.^20,23,31,32^ Similarly, human studies have also confirmed that the RSC has a direct dense structural connection to the HPC.^20,33^ Given that the RSC has stronger direct connections to the HPC than other parts of the PCC, it is reasonable to speculate that the RSC exhibits stronger functional connectivity with the HPC than other parts of the PCC, which is more closely linked to episodic memory processes. In fact, functional neuroimaging studies support this view and have proven that the RSC forms a critical gateway between the HPC and the other DMN regions to maintain communication between each other and to support episodic memory.^34,35^ More interestingly, a recent study has shown that the strength of connectivity between the RSC and HPC is related to medial parietal tau burden and that these tau and connectivity measures may play critical roles in Alzheimer’s disease progression.^36^ Collectively, these results suggest that RSC-HPC connections are crucial to understanding the evolution of cognitive dysfunction in Alzheimer’s disease. However, little is known about the pattern of functional connectivity between the RSC and HPC across different stages of the Alzheimer’s disease continuum.

Furthermore, it is acknowledged that the HPC is also heterogeneous and can be divided into subregions that have different functions, connectivity to other brain areas, and vulnerability to Alzheimer’s disease along its axis.^37-39^ The HPC can be subdivided along the longitudinal axis into the posterior (or dorsal) HPC and the anterior (or ventral) HPC.^40^ It has been suggested that the posterior HPC is particularly involved in episodic memory, whereas the anterior HPC is engaged in affective-related behaviours.^40,41^ Moreover, imaging studies have highlighted the fact that hippocampal subregions were described as being differentially affected in Alzheimer’s disease pathology.^38,39^ Thus, to gain a more accurate understanding of the RSC-HPC connectivity pattern across the Alzheimer’s disease spectrum, it is important to investigate the intrinsic connectivity between the RSC and the hippocampal subregions, as well as its changes in the onset and progression of Alzheimer’s disease. However, to date, no studies have evaluated the specificity of intrinsic connectivity between the RSC and the hippocampal subregions and its alteration across the different stages of the Alzheimer’s disease spectrum.

Therefore, the aim of the current study was to explore the specific intrinsic functional connectivity between the RSC and the hippocampal subregions in healthy adults and to characterize the alterations in functional connectivity between the RSC and the hippocampal subregions in aMCI and Alzheimer’s disease patients. It is hypothesized that the RSC exhibits stronger functional connectivity with the hippocampal subregions than other parts of the PCC. Furthermore, we hypothesized that altered functional connectivity between the RSC and the hippocampal subregions may demonstrate a trend of gradual decline from aMCI to Alzheimer’s disease.

## Materials and methods

### Subjects

The current study included 60 Alzheimer’s disease participants (31 females), 60 aMCI participants (31 females) and 60 normal controls (NCs) (34 females). All of the Alzheimer’s disease and aMCI participants included individuals who had consulted a memory clinic at Aerospace Center Hospital with memory complaints. The sex-matched NCs were recruited from the local community by using advertisements. Written informed consent was obtained from all of the participants or their legal guardians after the study had been fully explained. The authors assert that all of the procedures contributing to this research comply with the ethical standards of the relevant national and institutional committees on human experimentation and with the Helsinki Declaration of 1975, as revised in 2008. The study was approved by the Medical Research Ethics Committee of Aerospace Center Hospital.

All of the potential participants were screened by research clinicians according to the inclusion and exclusion criteria. Diagnoses of the Alzheimer’s disease and aMCI participants were made by two independent neurologists according to the standard criteria.^42,43^ The diagnosis of Alzheimer’s disease was based on the Diagnostic and Statistical Manual of Mental Disorders-V criteria for Alzheimer’s Dementia and the National Institutes on Aging and the Alzheimer’s Association (NIA-AA) on diagnostic guidelines for Alzheimer’s disease.^44^ Participants with aMCI were diagnosed according to Petersen’s clinical diagnostic criteria^43^ and the NIA-AA on diagnostic guidelines^42^ for aMCI due to Alzheimer’s disease. The inclusion criteria for NCs were as follows: (i) cognitively healthy individuals without memory complaints; (ii) mini-mental state examination (MMSE) score of 28 or higher; and (iii) clinical dementia rating (CDR)^45^ score of 0. The exclusion criteria for all of the participants were as follows: (i) a history of head trauma or surgery that causes cognitive impairment; (ii) a history of neurological or psychiatric disorders (such as Parkinson’s disease, severe anxiety, stroke or depression); and (iii) MRI contraindications.

### Image acquisition

Imaging data were acquired by the SIEMENS Trio 3-Tesla scanner (Siemens Medical Solutions, Erlangen, Germany). High-resolution T1-weighted anatomical images were acquired by using a 3D magnetization-prepared rapid gradient echo sequence with the following parameters: repetition time (TR) = 1900 ms, echo time (TE) = 2.2 ms, inversion time (TI) = 900 ms, flip angle (FA) = 9°, acquisition matrix = 224 × 256 × 176, and voxel size = 1 × 1 × 1 mm^3^. Resting-state fMRI datasets of the whole brain were axially obtained by using a T2*-weighted, multi-slice gradient echo planar imaging sequence with the following parameters: TR = 2000 ms, TE = 40 ms, FA = 90°, acquisition matrix = 64 × 64, field of view = 256 mm × 256 mm, slice thickness = 4 mm, gap = 1 mm, 28 slices and voxel size = 3.75 × 3.75 × 4 mm^3^. Suitable foam padding and headphones were used to limit head motion and to minimize scanner noise. During the MRI session, participants were instructed to hold still, keep their eyes closed, and to refrain from thinking of anything in particular. A simple questionnaire given after the scan confirmed that none of the subjects had fallen asleep.

### Image pre-processing

Pre-processing for rs-fMRI images was performed by using Statistical Parametric Mapping (SPM 12, University College London, London, UK; http://www.fil.ion.ucl.ac.uk/spm/software/spm12) and the CONN-fMRI functional connectivity toolbox v22a (http://www.nitrc.org/projects/conn^46^).

Briefly, the first 10 volumes were discarded to allow for signal stabilization. The remaining images were slice-time corrected (to the middle slice) and corrected for head motion (rigid body transformation by using six parameters). Based on the recording of head motion, 18 subjects who had images exceeding 2 mm translational movement or 2° rotational movement were excluded from further analysis (9 healthy participants, 6 aMCI participants and 3 Alzheimer’s disease participants were excluded for this reason). The head motion index (the average framewise displacement, FD^47^) was also calculated for each group, and the group differences were statistically analysed. To spatially normalize the fMRI data, the individual T1-weighted structural image was co-registered to the mean resting-state functional image. The resulting aligned T1 image was bias-corrected and segmented into grey matter, white matter (WM), and cerebrospinal fluid (CSF). The parameters that were obtained from this step were subsequently applied to the resting-state functional data (resampled to a voxel size of 3 × 3 × 3 mm^3^) during normalization to the MNI space. Afterwards, the functional data were spatially smoothed by using a 6 mm full width at half maximum Gaussian kernel. To better isolate distinct signal between the hippocampal subregions, the functional data with no spatial smoothing were also retained for the following analysis. Spurious sources of noise and signals from WM and CSF regions were estimated and regressed out by using the anatomical component base noise reduction strategy (aCompCor).^48^ The motion parameters were also regressed out. Finally, linear detrending and the recommended bandpass filter (0.009–0.08 Hz)^49^ were applied to reduce the effect of low-frequency drifts and high-frequency physiological noise.^50^

### Definition of regions of interest

**Figure 1:**
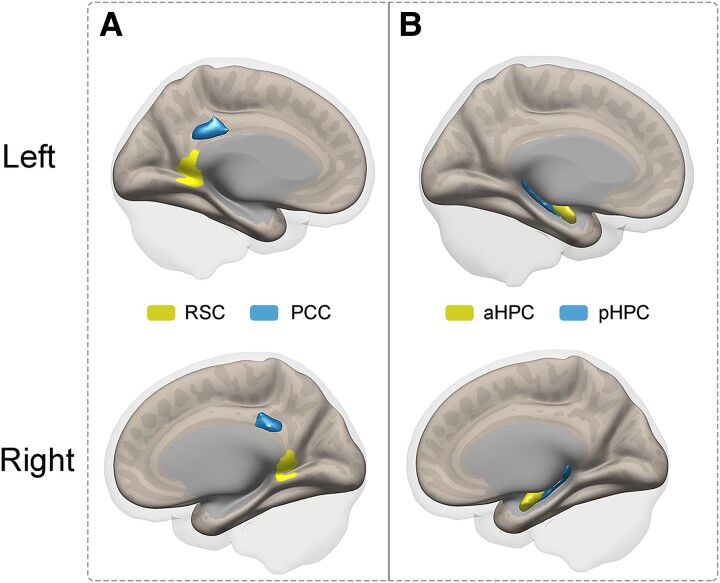
**Illustration of ROIs, which were obtained based on different probabilistic atlases.** (**A**) Yellow: RSC; blue: PCC. (**B**) Yellow: aHPC; blue: pHPC. RSC, retrosplenial cortex; PCC, posterior cingulate cortex; aHPC, anterior hippocampus; pHPC, posterior hippocampus.

In the current study, ROI-based analysis was performed, as we were particularly interested in the functional connections between the RSC and the hippocampal subregions. First, multiple brain regions were obtained based on different probabilistic atlases. The bilateral RSC, the bilateral other parts of PCC, and the bilateral anterior/posterior HPC were defined according to the Brainnetome Atlas (Brainnetome Atlas Viewer,^51^ vision 1.0.1, http://atlas.brainnetome.org/). The Brainnetome atlas, with 210 cortical and 36 subcortical subregions, provides a cross-validated atlas and contains information on the corresponding selected location with the different probability of being located within the targeted region.^51^ Therefore, the bilateral RSC and the bilateral other parts of PCC were further modified in location according to the Brainnetome atlas to verify the corresponding location with the probability of 90% of being located within the RSC or other parts of the PCC (Fig. 1A). The bilateral anterior/posterior HPC was confirmed to have a 50% probability of being located within the bilateral anterior/posterior HPC (Fig. 1B). When considering the abovementioned considerations, we ensured the highest overlap possible between our ROIs and the anatomical template, as well as the lowest overlap possible between other parts of the PCC and the RSC or between hippocampal subregions. Subsequently, to confirm the repeatability and reliability of the RSC or other parts of the PCC connectivity with the HPC, the spherical seed (7-mm radius) in the RSC (MNI coordinates: 2, −52, 16) (Supplementary Fig. S1A) and the same spherical seed in other parts of the PCC (MNI coordinates: 2, −30, 34) (Supplementary Fig. S1A) were also defined based on previously published literature.^24^

### Functional connectivity analysis

After data pre-processing, two sets of RSC or other parts of the PCC ROIs (according to the atlas and coordinates)-based resting-state connectivity analyses were performed to identify the RSC or other parts of the PCC functional connectivity with the HPC. The mean time series of the abovementioned ROIs were extracted from the denoised data (including smoothing and no spatial smoothing data) of each participant. Afterwards, RSC and other parts of the PCC ROI-based connectivity maps were generated for each participant by calculating semi-partial correlations, which allowed us to control for any potential signal bleed in and identify the unique contribution of a given ROI. Importantly, this approach allowed us to test hypotheses regarding specific functional patterns of the RSC and other parts of the PCC. Each semi-partial correlation map was converted into a Z value map by using Fisher’s r-to-z transformation to improve the normality of the correlation distribution. Subsequently, to examine the RSC or other parts of the PCC functional connectivity with the HPC, one-sample *t*-tests were separately performed for NC participants on the semi-partial correlation maps of the RSC and other parts of the PCC masked with the right or left hippocampal ROI images by using the Brainnetome Atlas. The significance level was set at a voxel level of *P* < 0.05, which was corrected for multiple comparisons by using familywise error (FWE). The functional connections between the RSC and the hippocampal subregions (including the bilateral anterior and posterior HPC) were also explored by using different masks of hippocampal subregions that were created above by following the same approach. The results of the RSC functional connectivity with the HPC and hippocampal subregions were saved as the masks for subsequent group-level analysis.

To determine significant intergroup differences, a voxelwise one-way ANOVA was performed to compare the functional connectivity difference between the RSC and the hippocampal subregions by using the abovementioned created masks across the Alzheimer’s disease, aMCI, and NC groups. The use of an F-test enabled us to compare the contribution of all six possible contrasts (pairwise comparison of the three groups). In this design, we were first interested in the main effects. If the main effect was significant, *post hoc* independent 2-sample *t*-tests were used to compare all three groups pairwise to each other, as the F-test is non-directional.

### Statistical analyses

One-way analysis of variance (ANOVA) and two-tailed χ^2^ tests were used to compare demographic features and neuropsychological scores among the three groups by using Statistical Package for Social Sciences version 22.0 (IBM SPSS 22.0, Chicago, IL, USA).

Group differences were compared by using one-way ANOVA within the aforementioned masks of the RSC functional connectivity with the HPC or hippocampal subregions after regressing out the influence of age, level of education, FD values, and total intracranial volume (TIV). The main effect map of the group was first generated, and Gaussian random field (GRF) theory was used for cluster-level multiple comparison correction (voxel *P-*value < 0.001; Cluster *P-*value < 0.05). *Post hoc* comparisons were used to identify meaningful RSC-HPC/HPC subregion functional connectivity differences among the three groups within the corresponding specific mask after controlling for the effects of age, education years, FD values and TIV values. The significance level was set with GRF corrected at *P* < 0.05.

To investigate the relationship between functional alterations and cognitive performance, the mean z values were extracted when statistically significant group differences were observed. Afterwards, Pearson’s correlation analysis was conducted to compute the correlation between behavioural data and neuroimaging indicators after controlling for the effects of age, sex and years of education. A value of *P* < 0.05 was considered to be statistically significant. Moreover, to investigate the classification ability (NC, aMCI and Alzheimer’s disease from each other) of all of the meaningful biomarkers in combination, receiver operating characteristic (ROC) analyses were performed by using SPSS software.

## Results

### Demographic and neuropsychological assessment

After careful evaluation of all available data, 18 participants (3 Alzheimer’s disease participants, 6 MCI participants, and 9 NCs) with poor image quality were excluded from the present study. When comparing the average FD values across different groups by using the remaining data, no group differences were found among the three groups (*F*_(2, 159)_ = 1.29, *P* = 0.28). Demographic data and behavioural assessments of 180 participants in the three groups can be found in Table 1. Participants in the Alzheimer’s disease, aMCI and NC groups were well matched for sex but differed significantly in age (*F*(2,177) = 4.11, *P* = 0.018). *Post hoc* analyses demonstrated that aMCI participants were older (*P* = 0.020) than NCs. Years of education (*F*(2,177) = 8.36, *P* < 0.001) were significantly lower in the Alzheimer’s disease (*P* < 0.001) and aMCI (*P* = 0.033) groups than in the NC group. Thus, age and years of education were included as covariates of no interest. As expected, there were significant differences across the three groups in cognitive performance. Compared with NCs, the aMCI group and the Alzheimer’s disease group both showed lower MMSE and CDR scores (Bonferroni corrected for *post hoc*, all *P* < 0.001). In addition, the Alzheimer’s disease group also showed significantly lower MMSE and CDR scores (Bonferroni corrected for *post hoc*, all *P* < 0.001) than the aMCI group.

**Table 1:** Demographic and neuropsychological assessments of participants

	AD (*n* = 60)	aMCI (*n* = 60)	NC (*n* = 60)	*P-*value
Age, years	66.07 (8.99)	69.40 (8.86)^a^	65.02 (8.39)	**0.018** *
Sex, males/females	29/31	29/31	26/34	0.82^#^
Education, years	8.57 (3.97)^b^	9.52 (3.67)^a^	11.18 (2.91)	**<0.001** *
MMSE scores	14.90 (6.52)^b,c^	24.07 (3.77)^a^	28.02 (2.31)	**<0.001** *
CDR, (0, 0.5, 1–2)	0.5 = 1, 1–2 = 59^b,c^	0.5 = 60^a^	0 = 60	**<0.001** ^#^
Framewise displacement^d^	0.19 (0.087)	0.18 (0.081)	0.16 (0.064)	0.28*

Results are given as means (standard deviation, SD). One-way ANOVA and χ^2^ analyses were applied to test for group differences, statistical significance level was set at *P* < 0.05 (two-tailed). Significant *P*-values were in bold.

MMSE, mini-mental state examination; CDR, clinical dementia rating; AD, Alzheimer’s disease; aMCI, amnestic mild cognitive impairment; NC, normal controls.

^a^ *Post hoc* pairwise comparisons showed a significant group difference between aMCI and NC (Bonferroni corrected *P* < 0.05).

^b^ *Post hoc* pairwise comparisons showed a significant group difference between AD and NC (Bonferroni corrected *P* < 0.05).

^c^ *Post hoc* pairwise comparisons showed a significant group difference between AD and aMCI (Bonferroni corrected *P* < 0.05).

^d^Eighteen subjects were not included due to poor image quality (AD = 3, aMCI = 6, NC = 9).

^*^The *P-*value was obtained using One-way ANOVA.

^#^The *P-*value was obtained using χ^2^ test with multiple comparison.

### Comparison of functional connectivity between the RSC and the hippocampal subregions

**Figure 2:**
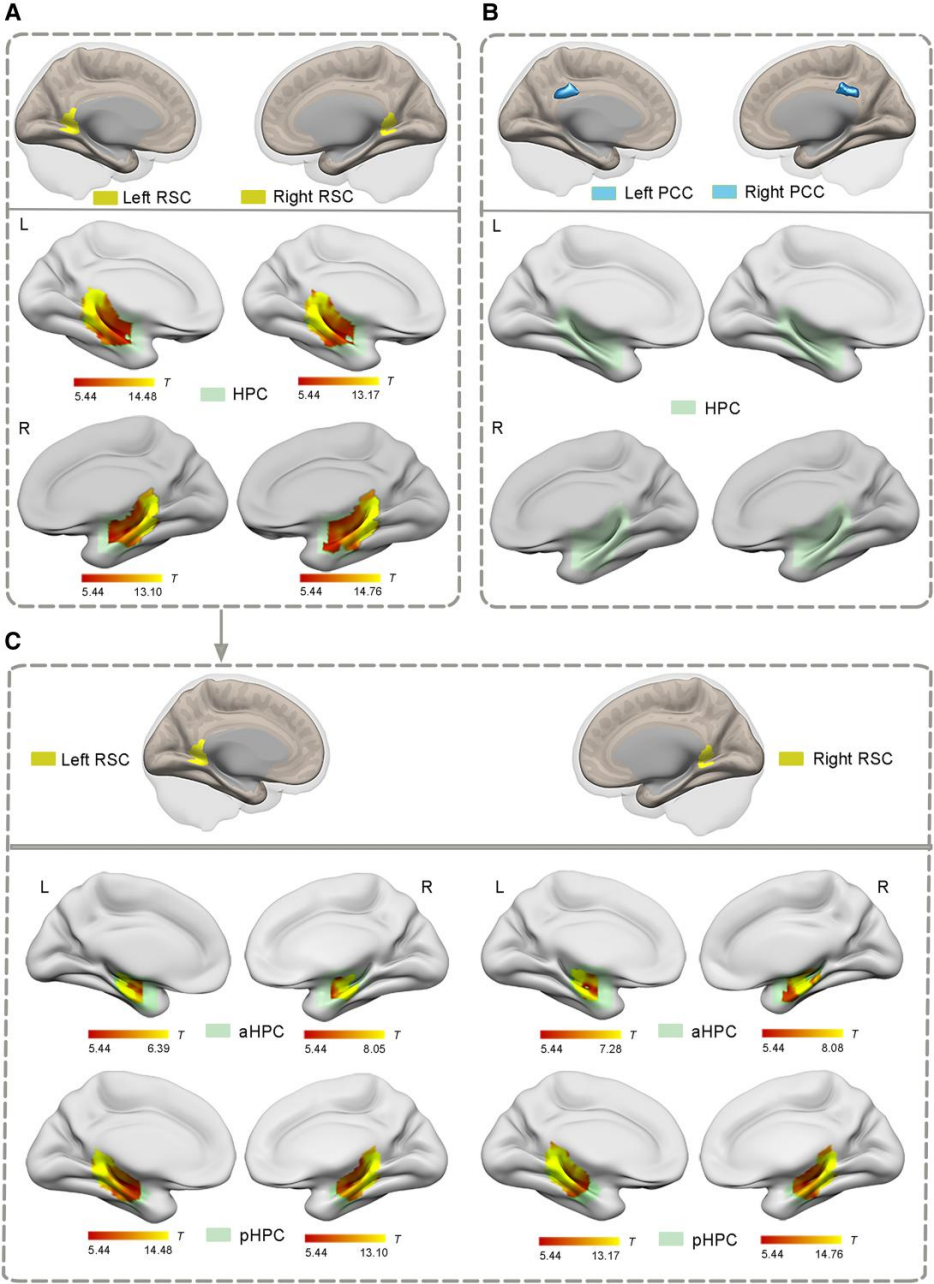
**The one-sample *t*-test of functional connectivity patterns of the bilateral RSC and other parts of the PCC with the HPC, as well as functional connectivity between the bilateral RSC and the hippocampal subregions in the NC group.** (**A**) The bilateral RSC (yellow colour) both showed significantly positive functional connectivity with the bilateral HPC (shown in warm colours; using the one-sample *t*-test in the NC group (*n* = 51), *P* < 0.05, FWE corrected). (**B**) No regions showing positive functional connectivity between the bilateral other parts of PCC (blue colour) and the HPC. (**C**) The bilateral RSC (yellow colour) showing significantly positive functional connectivity with the bilateral aHPC and pHPC (shown in warm colours; using the one-sample *t*-test in the NC group (*n* = 51), *P* < 0.05, FWE corrected). RSC, retrosplenial cortex; PCC, posterior cingulate cortex; HPC, hippocampus; aHPC, anterior hippocampus; pHPC, posterior hippocampus; L, left; R, right.

Figure 2A illustrate z-maps of the RSC functional connectivity with the HPC, as demonstrated by the one-sample *t*-test in the NC group. The results demonstrated that the left RSC and the right RSC both showed significantly positive functional connectivity with the bilateral HPC (*P* < 0.05, FWE corrected; Fig. 2A and Table 2), whereas the bilateral other parts of PCC showed no significant functional connectivity with the bilateral HPC (Fig. 2B). The similar functional connectivity patterns of the RSC and other parts of the PCC were also validated by using RSC-seed and other parts of the PCC-seed connectivity (Supplementary Fig. S2). Furthermore, Fig. 2C shows z-maps of functional connectivity between the RSC and the hippocampal subregions in the NCs, and the results demonstrated that the left RSC and the right RSC both showed significantly positive functional connectivity with the bilateral anterior HPC and the bilateral posterior HPC (*P* < 0.05, FWE corrected; Fig. 2C and Table 2). The above results were also validated by using functional data with no spatial smoothing (Supplementary Fig. S3 and Table S1).

**Table 2:** Functional connectivity between the RSC and the hippocampal subregions in the NC group

ROIs	Brain regions	Side	Cluster size	*T* value	Peak MNI coordinates		
					X	Y	Z
Left RSC	HPC	L	344	14.48	−12	−38	−2
		R	372	13.10	16	−38	0
	aHPC	L	67	6.39	−24	−18	−22
		R	68	8.05	24	−18	−18
	pHPC	L	277	14.48	−12	−38	−2
		R	290	13.10	16	−38	0
Right RSC	HPC	L	300	13.17	−10	−38	0
		R	353	14.76	16	−40	2
	aHPC	L	68	6.66	−24	−20	−16
		R	101	8.53	24	−18	−18
	pHPC	L	232	13.17	−10	−38	0
		R	260	14.76	16	−40	2

The RSC showed significant functional connectivity with the hippocampal subregions in the NC group (*P* < 0.05, FWE corrected).

RSC, retrosplenial cortex; HPC, hippocampus; aHPC, anterior hippocampus; pHPC, posterior hippocampus; L, left; R, right; MNI, Montreal neurological institute; NC, normal controls.

**Figure 3:**
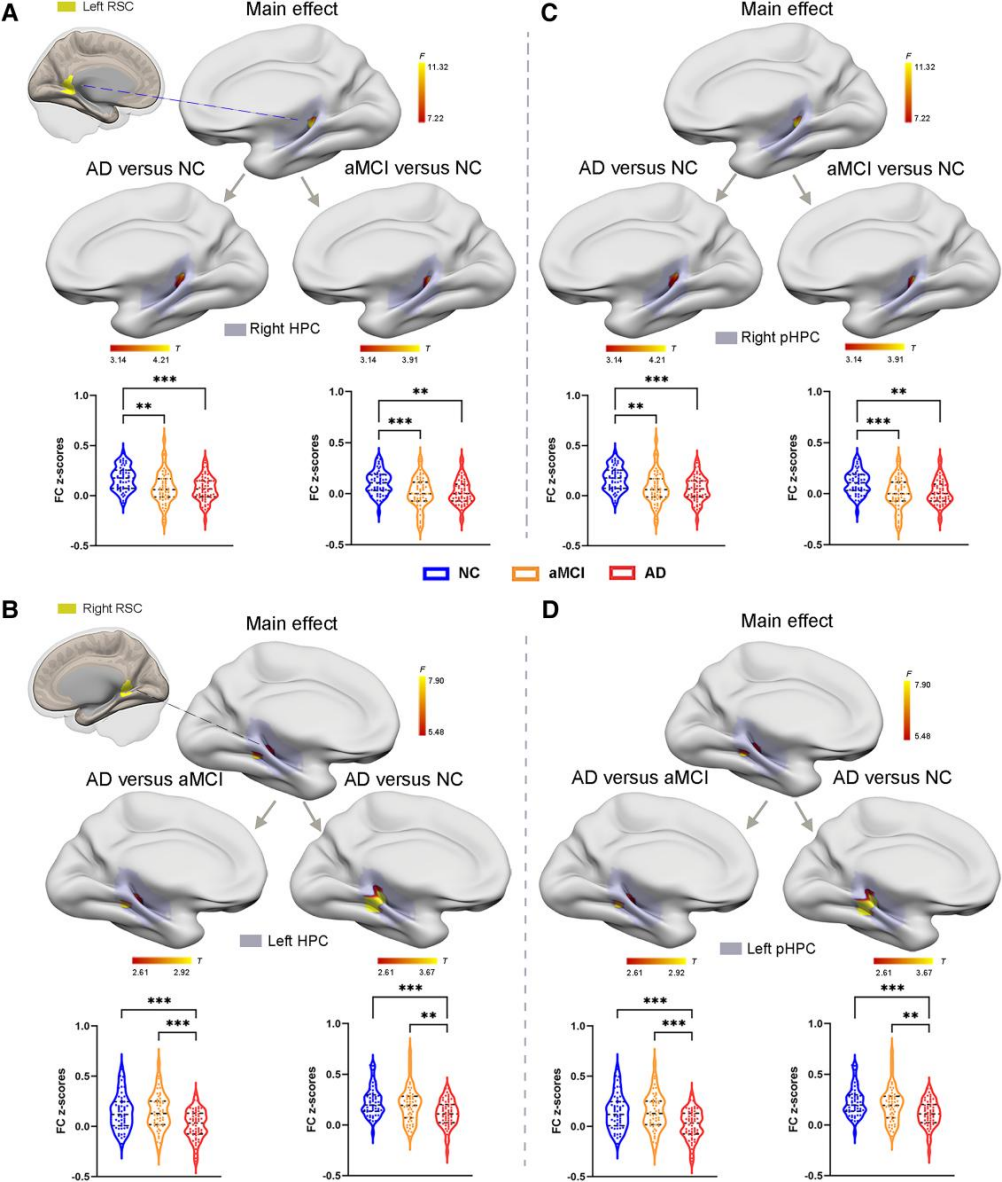
**Group differences in functional connectivity of the bilateral RSC with the HPC and the hippocampal subregions among the AD, aMCI and NC groups.** (**A**) Regions showing altered functional connectivity between the left RSC and the right HPC among the three groups (mean ± SD are reported from *n* = 162 participants, one-way ANOVAs, with Bonferroni’s method for pairwise comparisons, the blue violin plots represent the NC group, the brown violin plots represent the aMCI group and the red violin plots represent the AD group). (**C**) Regions showing altered functional connectivity between the left RSC and the right posterior HPC among the three groups (mean ± SD are reported from *n* = 162 participants, one-way ANOVAs, with Bonferroni’s method for pairwise comparisons, the blue violin plots represent the NC group, the brown violin plots represent the aMCI group and the red violin plots represent the AD group). (**B**) Regions showing altered functional connectivity between the right RSC and the left HPC among the three groups (mean ± SD are reported from *n* = 162 participants, one-way ANOVAs, with Bonferroni’s method for pairwise comparisons, the blue violin plots represent the NC group, the brown violin plots represent the aMCI group and the red violin plots represent the AD group). (**D**) Regions showing altered functional connectivity between the right RSC and the left posterior HPC among the three groups (mean ± SD are reported from *n* = 162 participants, one-way ANOVAs, with Bonferroni’s method for pairwise comparisons, the blue violin plots represent the NC group, the brown violin plots represent the aMCI group and the red violin plots represent the AD group). Violin plots displayed mean resting-state functional connectivity z scores for the AD, aMCI and NC groups. Each datapoint represents mean functional connectivity z-score of each individual. The z scores were extracted from regions showing significant group effects by ANOVA and further performed *post hoc* pairwise comparisons. ∗Statistically significant at the 0.05 level; ∗∗Statistically significant at the 0.01 level; ∗∗∗Statistically significant at the 0.001 level. RSC, retrosplenial cortex; HPC, hippocampus; pHPC, posterior hippocampus; AD, Alzheimer’s disease; aMCI, amnestic mild cognitive impairment; NC, normal controls; FC, functional connectivity.

Group differences in connectivity among Alzheimer’s disease, aMCI and NC participants are illustrated in Fig. 3. ANOVA showed significant alterations across the three groups concerning RSC functional connectivity with the HPC. *Post hoc* pairwise 2-sample *t*-tests demonstrated that compared to the NCs, the Alzheimer’s disease group and the aMCI group both exhibited significantly decreased functional connectivity between the left RSC and the right HPC (Fig. 3A and Table 3). In addition, compared to both the NCs and aMCI participants, Alzheimer’s disease patients showed decreased functional connectivity between the right RSC and the left HPC (Fig. 3B and Table 3). At the hippocampal subregion level, the Alzheimer’s disease group and the aMCI group both had significantly decreased functional connectivity from the left RSC to the right posterior HPC (Fig. 3C and Table 3). Alzheimer’s disease patients also displayed significantly decreased functional connectivity from the right RSC to the left posterior HPC (Fig. 3D and Table 3) relative to NCs and aMCI participants. The similar functional connectivity results between the RSC and hippocampal subregions were also validated by using functional data with no spatial smoothing (Supplementary Fig. S4 and Table S2). Except for the above results, there were no other significant differences in functional connectivity among the three groups.

**Table 3:** Comparison of functional connectivity among the HC, aMCI and AD groups

Comparison	Brain regions	Side	Cluster size	*F/T* value	Peak MNI coordinates		
					X	Y	Z
Left RSC							
Main effect	HPC	R	24	11.32	28	−40	0
AD < NC	HPC	R	32	4.21	28	−40	0
aMCI < NC	HPC	R	16	3.91	28	−40	0
Main effect	pHPC	R	24	11.32	28	−40	0
AD < NC	pHPC	R	32	4.21	28	−40	0
aMCI < NC	pHPC	R	16	3.91	28	−40	0
Right RSC^a^							
Main effect	HPC	L	11	7.90	−32	−40	−6
AD < NC	HPC	L	34	3.67	−32	−40	−6
AD < aMCI	HPC	L	19	2.92	−32	−40	−6
Main effect	pHPC	L	11	7.90	−32	−40	−6
AD < NC	pHPC	L	34	3.67	−32	−40	−6
AD < aMCI	pHPC	L	19	2.92	−32	−40	−6

Brain regions showed significant differences in functional connectivity among the three groups (Gaussian random field (GRF) corrected (voxel *P* < 0.001, cluster *P* < 0.05)).

RSC, retrosplenial cortex; HPC, hippocampus; pHPC, posterior hippocampus; L, left; R, right; MNI, Montreal neurological institute; NC, normal controls; aMCI, amnestic mild cognitive impairment; AD, Alzheimer’s disease.

^a^Gaussian random field (GRF) corrected (voxel *P* < 0.005, cluster *P* < 0.05).

### Pearson correlation analysis and ROC analysis

**Figure 4:**
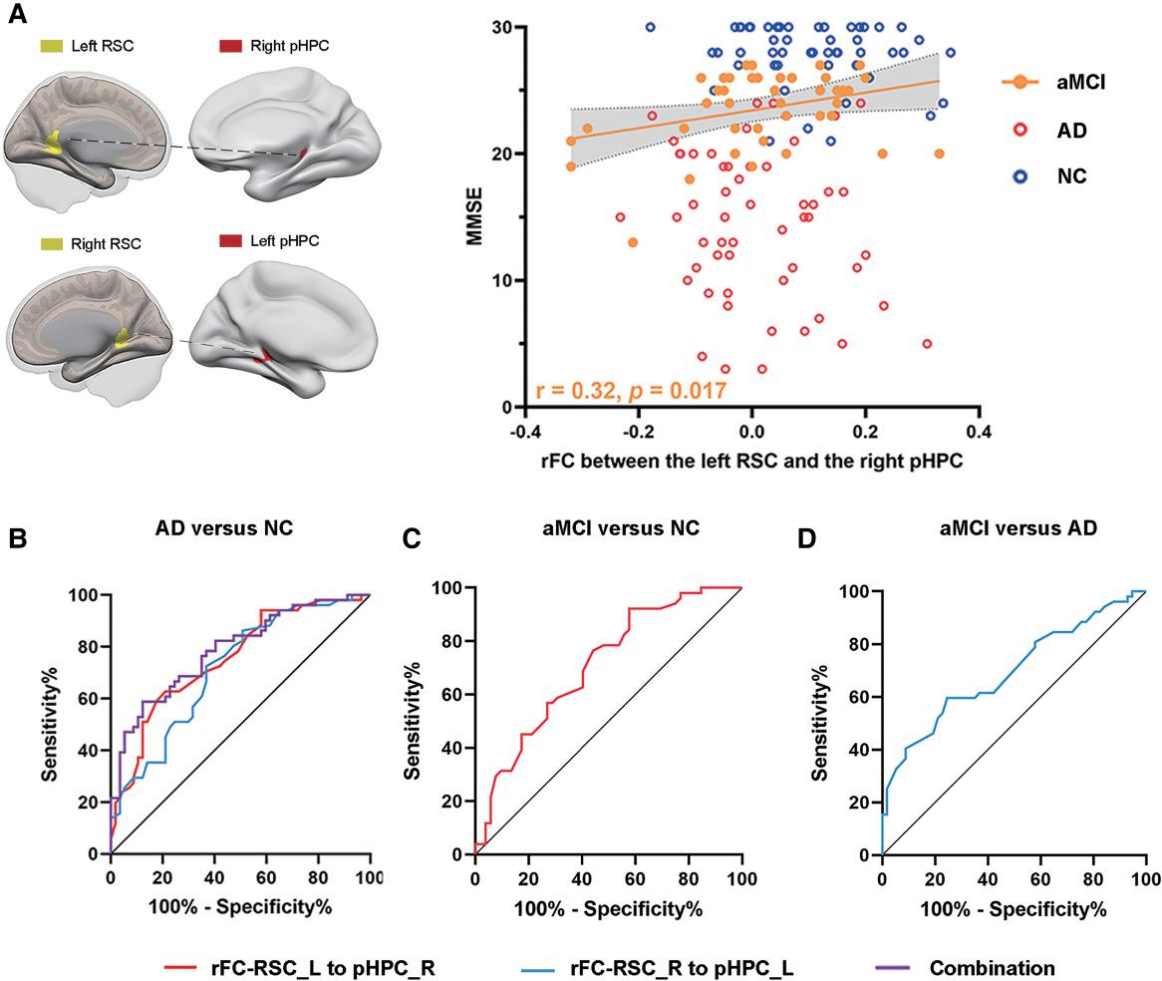
**Pearson correlation analysis and ROC analysis across three groups.** Brain maps of related areas are showed in the figure and coloured areas represent their locations. Dashed lines are for illustrating purpose. (**A**) The connectivity strengths between the left RSC and right posterior HPC had correlations with the MMSE scores in the aMCI group (using Pearson correlation analysis in the aMCI group (*n* = 54), the brown dots represent the distribution of data in the aMCI group, the red circles and blue circles represent the distribution of data in the AD and NC groups, respectively). (**B**) ROC curve presenting the classification of AD versus NC (the AUC values of functional connectivity between the left RSC and right pHPC, as well as functional connectivity between the right RSC and left pHPC, were 0.75 and 0.72, respectively. The AUC value of the combined parameter was 0.79); (**C**) ROC curve presenting the classification of aMCI versus NC (the AUC value of functional connectivity between the left RSC and right pHPC was 0.71); (**D**) ROC curve presenting the classification of aMCI versus AD (the AUC value of functional connectivity between the right RSC and left pHPC was 0.70). RSC, retrosplenial cortex; pHPC, posterior hippocampus; AD, Alzheimer’s disease; aMCI, amnestic mild cognitive impairment; NC, normal controls; MMSE, mini-mental state examination; rFC, resting-state functional connectivity; AUC, area under the curve; L, left; R, right.

Pearson correlation analysis was conducted between altered functional connectivity and neuropsychological scales in the Alzheimer’s disease and aMCI groups. The significant associations are summarized in Fig. 4. The connectivity strengths between the left RSC and right posterior HPC showed a positive correlation with the MMSE scores (r = 0.32, *P* = 0.017) in the aMCI group (Fig. 4A). There was no significant correlation between abnormal functional connectivity values and neuropsychological scales in the Alzheimer’s disease group (*P* > 0.05).

In the Alzheimer’s disease and NC groups, the area under the curve (AUC) values of functional connectivity between the left RSC and right posterior HPC, as well as functional connectivity between the right RSC and left posterior HPC, were 0.75 and 0.72, respectively. The AUC value of the combined parameter (combination using both of the abovementioned functional connectivity parameters) was 0.79, with a corresponding sensitivity and specificity of 59 and 88% (all *P* < 0.05, Fig. 4B). In the aMCI and NC groups, the AUC value of functional connectivity between the left RSC and right posterior HPC was 0.71, with a corresponding sensitivity and specificity of 69 and 60% (*P* < 0.05, Fig. 4C). In the aMCI and Alzheimer’s disease groups, the AUC value of functional connectivity between the right RSC and left posterior HPC was 0.70, with a corresponding sensitivity and specificity of 60 and 77% (*P* < 0.05, Fig. 4D).

## Discussion

In this study, ROIs derived from the atlas and coordinates were used to investigate the specific intrinsic functional connectivity between the RSC and the HPC subregions (including anterior and posterior HPC subregions) in healthy adults and to assess how its distinct patterns of functional connectivity may demonstrate selective vulnerability associated with progression along the spectrum from healthy aging to Alzheimer’s disease. A number of interesting results emerged from the current study. First, it was the RSC (rather than other parts of the PCC) that was mainly functionally connected with the HPC. Second, when evaluating the RSC being functionally connected with any of the hippocampal subregions, we found widespread intrinsic functional connectivity of the RSC with all of the anterior–posterior HPC subregions. Third, functional connectivity between the RSC and the HPC subregions was not uniformly affected in aMCI and Alzheimer’s disease; rather, it selectively impacted the connectivity between the RSC and the contralateral posterior HPC, thus indicating a trend of gradual decline from aMCI to Alzheimer’s disease. Furthermore, our results demonstrate that diminished functional connectivity between the left RSC and right posterior HPC was correlated with clinical disease severity in aMCI patients. These findings are critical for understanding adaptations in the connectivity pattern of RSC-HPC subregions during the progression from aMCI to Alzheimer’s disease.

Previous evidence suggests that the RSC is typically included as part of the PCC and involved in a broad range of cognitive functions, including episodic memory, navigation, imagination and future planning.^20^ Animal and human studies have also demonstrated that the PCC is a cytoarchitectonically distinct and functionally heterogeneous region, and it is important to differentiate the RSC from other parts of the PCC because of many structural and functional variations.^20,23,31^ Furthermore, previous anatomical and rs-fMRI studies have demonstrated that the RSC has dense anatomical connections to the HPC and that the RSC (in conjunction with the HPC) is important to episodic memory.^20,31-33^ Thus, the PCC-HPC interaction reported by previous human studies^20,52^ may reflect the interconnectivity between the RSC and the HPC. Herein, our study investigating the intrinsic functional connectivity between the RSC and the HPC is consistent with these findings, thus demonstrating heterogeneity in the degree of connectivity between the HPC and different parts of the PCC, with extremely greater functional connectivity of the RSC with the HPC compared with other parts of the PCC. Our result of extremely greater RSC-HPC functional connectivity also adds to growing evidence that the RSC forms a critical gateway between the HPC and DMN regions to enable communication between the HPC and cortical DMN.^34,35^

It is important to note that previous studies have observed connectivity variation along the anterior–posterior axis of the HPC.^41,53^ Two gradients of functional connectivity that ran in the anterior/posterior and medial/lateral directions were identified.^54^ The anterior HPC was primarily connected with temporal regions and the orbitofrontal cortex, whereas connectivity of the posterior HPC was more highly connected in the medial and lateral parietal cortex, including the RSC.^53,55^ To quantitatively determine spatial overlap between the areas, which had significant connectivity with the RSC and the boundaries of the anterior HPC and posterior HPC, ROI (the significant connectivity results) to ROI (anterior HPC and posterior HPC masks) analysis was conducted. As shown in Supplementary Fig. S5, functional connectivity between the significant connectivity results and the ROI boundaries of posterior HPC was significantly higher than that of connectivity with the ROI boundaries of anterior HPC. Moreover, functional connectivity between the RSC and the posterior HPC was significantly greater than functional connectivity between the RSC and the anterior HPC (Supplementary Fig. S6). In conjunction with these findings, our results showed that functional connectivity between the RSC and the posterior HPC was greater and more extensive than functional connectivity between the RSC and the anterior HPC, and the significant connectivity between RSC and HPC had higher spatial overlap with posterior HPC than that of anterior PHC.

The RSC and HPC have been well established to play a critical role in episodic memory.^20,56^ Therefore, both regions are known to be the earliest brain areas to be preferentially affected in the early stages of Alzheimer’s disease.^14,25^ Relatedly, decreased functional connectivity between the PCC/RSC and HPC has been broadly reported in subjects with MCI and Alzheimer’s disease.^6-9^ In accordance with prior studies, our results also demonstrated reduced functional connectivity between the RSC and HPC in aMCI and Alzheimer’s disease patients compared to NC participants in a gradient manner. Interestingly, our results only demonstrated disrupted contralateral coupling of the RSC/HPC in aMCI and Alzheimer’s disease patients relative to NCs. Functional connectivity is a statistical concept and it identifies significant deviations between the activity of neuronal populations regardless of whether these elements are connected by direct structural links or not.^57^ Although this still remains speculative, it may be related to asymmetric rather than lateralized atrophy in Alzheimer’s disease or the inherent lower connections of contralateral brain regions.^58,59^ Evidence suggests that Aβ accumulation preferentially starts in the PCC, i.e. in several of the core regions of the DMN, which has been linked to network failure in the posterior DMN.^60,61^ Additionally, previous literature has suggested that patterns of functional connectivity are associated with tau deposition and that tau spreads through functional connectivity, which closely resembles the observed pattern of tau deposition in the brain.^62,63^ Although recent study suggests heterogeneity in patterns of spatiotemporal trajectories of tau deposition in patients with Alzheimer’s disease and four distinct patterns are identified including limbic-predominant, medial temporal lobe-sparing, posterior and lateral temporal patterns,^64^ these subtypes all include medial parietal lobe as a vulnerable region. Furthermore, functional connectivity between the RSC and HPC is associated with medial parietal tau, thus suggesting that tau in the HPC spreads directly to the medial parietal lobe via connectivity with the RSC.^36^ The combination of greater medial parietal lobe tau accumulation and greater RSC-HPC strength is also related to episodic memory performance.^36^ Taken together, it is therefore plausible that disrupted functional connectivity between the RSC and HPC may partially underlie early episodic memory deficits in the Alzheimer’s disease spectrum via tau spreading and accumulation.

More importantly, the current functional connectivity results suggested that functional connectivity between the RSC and HPC was selectively affected in aMCI and Alzheimer’s disease patients, which demonstrated solely reduced functional connectivity between the RSC and the posterior HPC. To our knowledge, there are no studies focusing on the altered connectivity between the RSC and the hippocampal subregions along the Alzheimer’s disease continuum, which actually seems to be of vital importance to understanding episodic memory deficits in prodromal Alzheimer’s disease. In terms of function, the dominant viewpoint is that the anterior HPC is engaged in affective-related behaviours and coarse gist-like memory, whereas the posterior HPC is particularly implicated in detailed episodic memory, navigation and visuospatial memory.^40,41^ Our correlation results further demonstrated that reduced functional connectivity between the left RSC and right posterior HPC was associated with cognitive abnormalities in aMCI patients. In the current study, aMCI was mainly characterized by isolated memory impairment. Thus, this finding may also suggest that disrupted functional connectivity between the RSC and the posterior HPC underlies behavioural manifestations of episodic memory caused by underlying Alzheimer’s disease pathology. In addition, another important finding in this study was that functional connectivity between the RSC and the hippocampal subregions contributed to the classification among NC, aMCI, and Alzheimer’s disease subjects. To discriminate Alzheimer’s disease from NC, the combination of the left RSC-right posterior HPC and the right RSC-left posterior HPC had a sensitivity of 59% and specificity of 88%. To discriminate aMCI from Alzheimer’s disease, the functional connectivity between the left RSC and right posterior HPC had a sensitivity of 60% and specificity of 77%. All of the results demonstrated the advantages of the functional alterations of the RSC and the hippocampal subregions in studying prodromal stages of Alzheimer’s disease and provided a potential mechanism for conversion from aMCI to Alzheimer’s disease.

## Limitations

When considering these interesting findings, some limitations of this study should be taken into consideration. First, age and educational level were not well matched in these three groups. Although we included age, gender and education as covariates in the process of statistical analyses to account for this putative confound, we cannot completely rule out potential bias due to these effects. Future studies are needed to validate our results and to exclude possible confounding effects on any observed differences in our current data. Second, cognitive tasks were not used to evaluate episodic memory ability. Although the MMSE score could be used to assess episodic memory in aMCI subjects due to isolated memory impairment, we cannot determine episodic memory impairment in the Alzheimer’s disease group. This may also explain why we did not observe any association between the MMSE score and the functional connectivity between the RSC and the hippocampal subregions. Third, this study did not include information on the biomarkers of Alzheimer’s disease (such as amyloid-β (Aβ42), total tau (T-tau) and phosphorylated tau (*P*-tau)) to confirm the diagnosis of Alzheimer’s disease. Thus, we could not rule out other concomitant pathology (including Lewy bodies, phosphorylated TDP-43 (p-TDP-43) positive neuronal cytoplasmic inclusions and vascular pathology), which can cause Alzheimer’s disease symptoms. Fourth, resting-state fMRI data is commonly acquired in one of three different conditions, eyes closed, eyes open and eyes fixated on a target. Many previous studies have demonstrated that the eyes fixated on a target condition may be a better choice for functional connectivity studies that aim to generate greater reliability and consistency in core functional networks.^65,66^ Future studies should optimize the data acquisition and collect resting-state data with eyes open and fixated on a target. Last, the current study did not investigate RSC functional abnormalities by using a dynamic functional connectivity approach, which is considered to be a powerful supplement to static functional connectivity and may more sensitively reflect abnormal connectivity.^67,68^ In the future, we will focus more attention on dynamic changes in connectivity patterns between the RSC and the HPC subregions to enable a more comprehensive understanding of adaptations in the connectivity pattern of RSC-HPC subregions during the progression of Alzheimer’s disease continuum.

## Conclusions

Overall, our current study provides evidence that the functional organization of the total PCC demonstrates distinct functional connectivity patterns and that the RSC shows extremely greater functional connectivity with the HPC than other parts of the PCC. Furthermore, analysis of the connectivity patterns between the RSC and the HPC subregions in the Alzheimer’s disease spectrum demonstrated selective functional connectivity disruptions between the RSC and the contralateral posterior HPC with a trend of gradual decline from aMCI to Alzheimer’s disease. Abnormal functional connectivity between the left RSC and right posterior HPC is associated with disease severity and episodic memory impairments in aMCI subjects, which suggests a relationship between intrinsic functional disintegration between these regions and subsequent cognitive manifestations. Taken together, these findings emphasize the importance of functional heterogeneity of the total PCC, thus suggesting that distinct functional abnormalities between the RSC and the HPC subregions are related to episodic memory deficits in the course of Alzheimer’s disease progression.

## Supplementary Material

fcae476_Supplementary_Data

## Data Availability

The data, which support this study, is not publicly available but may be provided upon reasonable request.
