# Supplementary material for: Aberrant functional connectivity between the retrosplenial cortex and hippocampal subregions in amnestic mild cognitive impairment and Alzheimer’s disease
Source: Brain Commun. 2024 Dec 31;7(1):fcae476. doi: 10.1093/braincomms/fcae476 (PMC11733685; doi:10.1093/braincomms/fcae476)
Supplement: fcae476_Supplementary_Data [file fcae476_supplementary_data.pdf]

Aberrant functional connectivity between the retrosplenial cortex and hippocampal subregions in amnestic mild cognitive impairment and Alzheimer's disease

Supplemental Data:

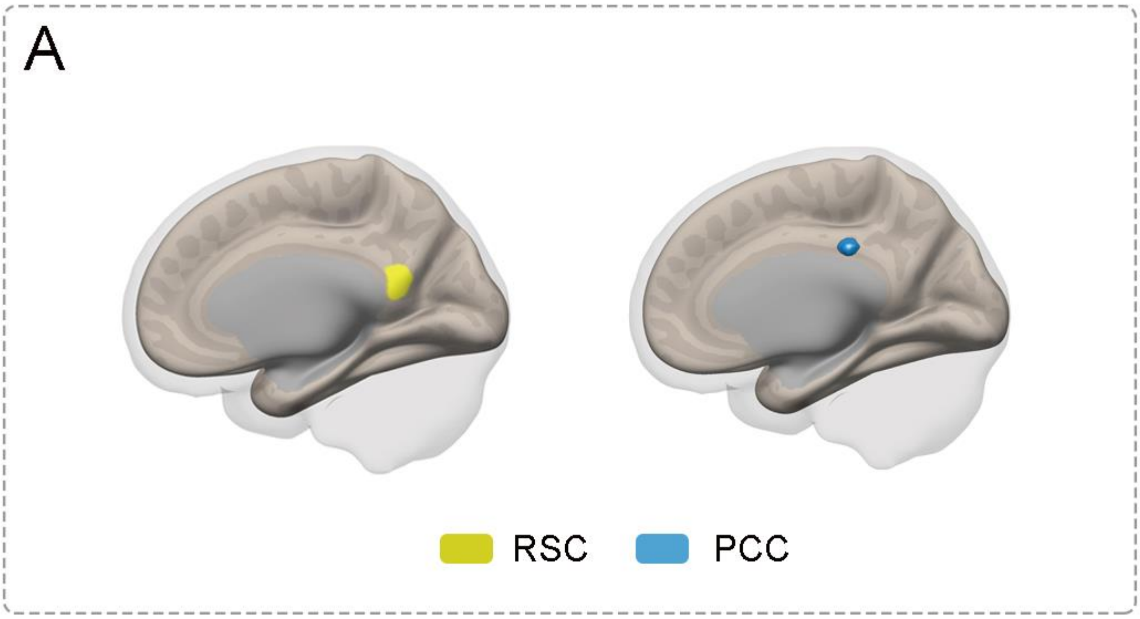

Supplementary Figure 1 Illustration of the ROIs. 7-mm retrosplenial cortex seed region in yellow (MNI coordinates: 2, -52, 16) and posterior cingulate cortex seed region in blue (MNI coordinates: 2, -30, 34). RSC, retrosplenial cortex; PCC, posterior cingulate cortex.

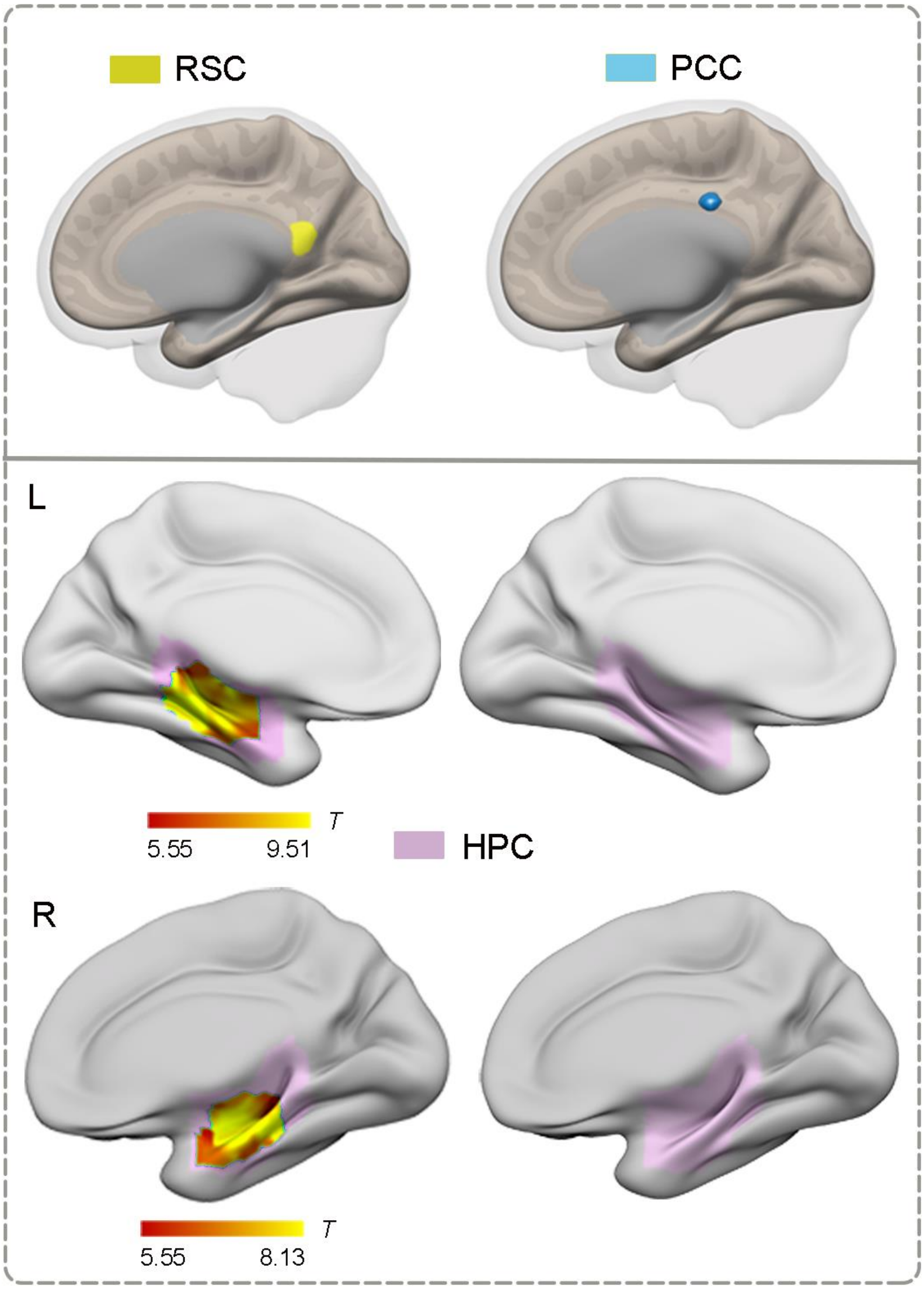

Supplementary Figure 2 The one-sample t test of seed-based functional connectivity patterns of the bilateral RSC and PCC with the HPC in the NC group. The bilateral RSC (yellow color) both showed significantly positive functional connectivity with the bilateral HPC (shown in warm colors; using the one-sample t test in the NC group (n = 51), $p < 0.05$, FWE corrected). No regions showing positive functional connectivity between the bilateral PCC (blue color) and the HPC (using the one-sample t test in the NC group (n = 51), $p < 0.05$, FWE corrected). RSC, retrosplenial cortex; PCC, posterior cingulate cortex; HPC, hippocampus.

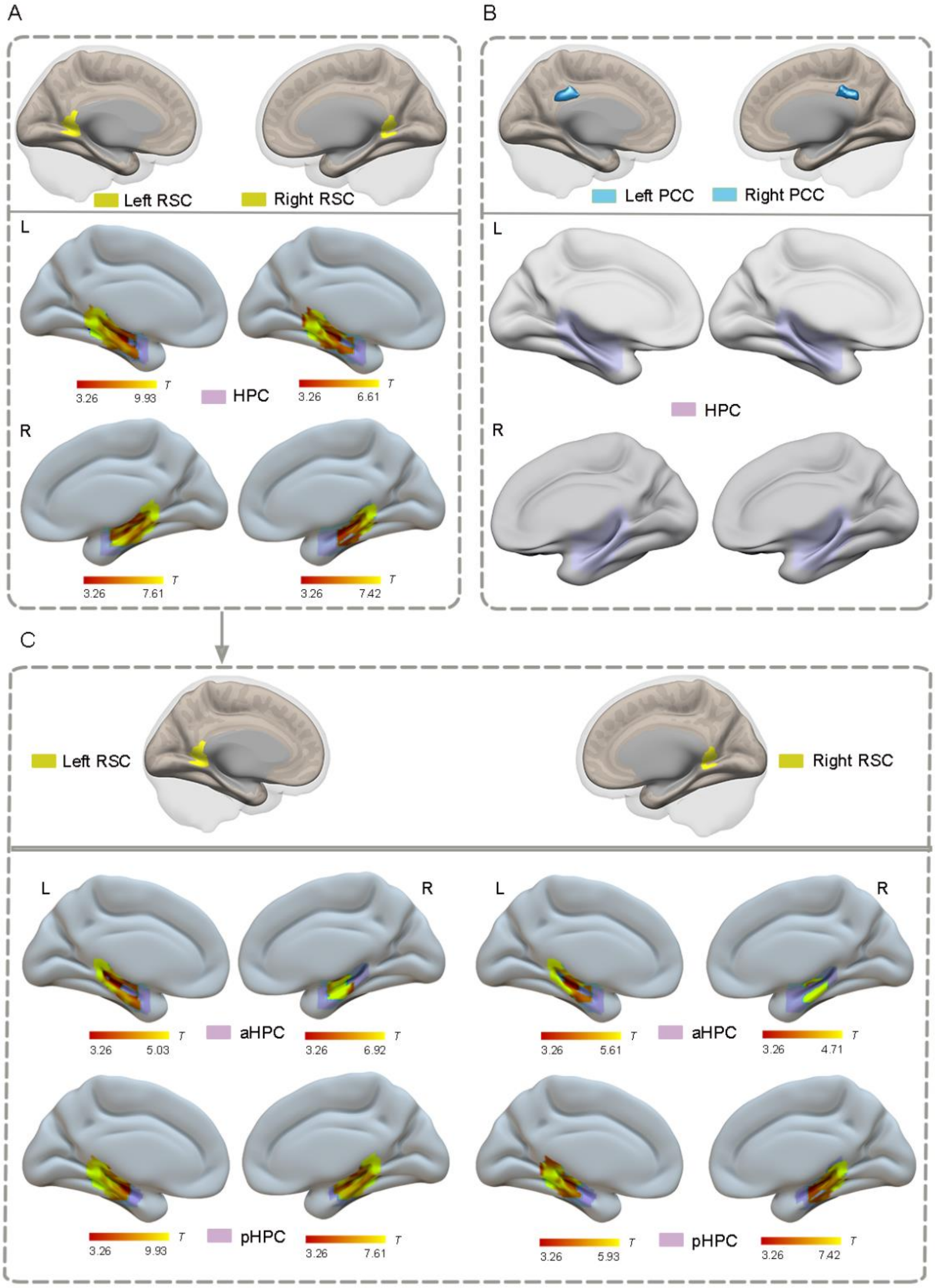

Supplementary Figure 3 The one-sample t test of functional connectivity patterns of the bilateral RSC and other parts of the PCC with the HPC, as well as functional connectivity between the bilateral RSC and the hippocampal subregions in the NC group with no smoothing, and semipartial correlations. (A) The bilateral RSC (yellow color) both showed significantly positive functional connectivity with the bilateral HPC (shown in warm colors; using the one-sample t test in the NC group (n = 51), $p < 0.05$, FWE corrected for multiple comparisons on cluster level). (B) No regions showing positive functional connectivity between the bilateral other parts of PCC (blue color) and the HPC. (C) The bilateral RSC (yellow color) showing

significantly positive functional connectivity with the bilateral aHPC and pHPC (shown in warm colors; using the one-sample t test in the NC group (n = 51), $p < 0.05$, FWE corrected for multiple comparisons on cluster level). RSC, retrosplenial cortex; PCC, posterior cingulate cortex; HPC, hippocampus; aHPC, anterior hippocampus; pHPC, posterior hippocampus.

Supplementary Table 1 Functional connectivity between the RSC and the hippocampal subregions in the NC group with no smoothing, and semipartial correlations

ROIs	Brain regions	Side	Cluster size	*T* value	Peak MNI coordinates		
					X	Y	Z
Left RSC	HPC	L	393	9.93	-12	-38	-2
		R	392	7.61	24	-40	-2
	aHPC	L	118	5.03	-24	-18	-22
		R	76	6.92	24	-18	-18
	pHPC	L	275	9.93	-12	-38	-2
		R	297	7.61	24	-40	-2
Right RSC	HPC	L	213	6.61	-10	-38	0
		R	122	7.42	16	-38	0
	aHPC	L	60	5.61	-24	-20	-16
		R	34	4.71	20	-26	-14
	pHPC	L	153	5.93	-24	-40	-6
		R	116	7.42	16	-38	0

Note: The RSC showed significant functional connectivity with the hippocampal subregions in the NC group ($p < 0.05$, FWE corrected for multiple comparisons on cluster level).

Abbreviations: RSC, retrosplenial cortex; HPC, hippocampus; aHPC, anterior hippocampus; pHPC, posterior hippocampus; L, left; R, right; MNI, Montreal neurological institute; NC, normal controls.

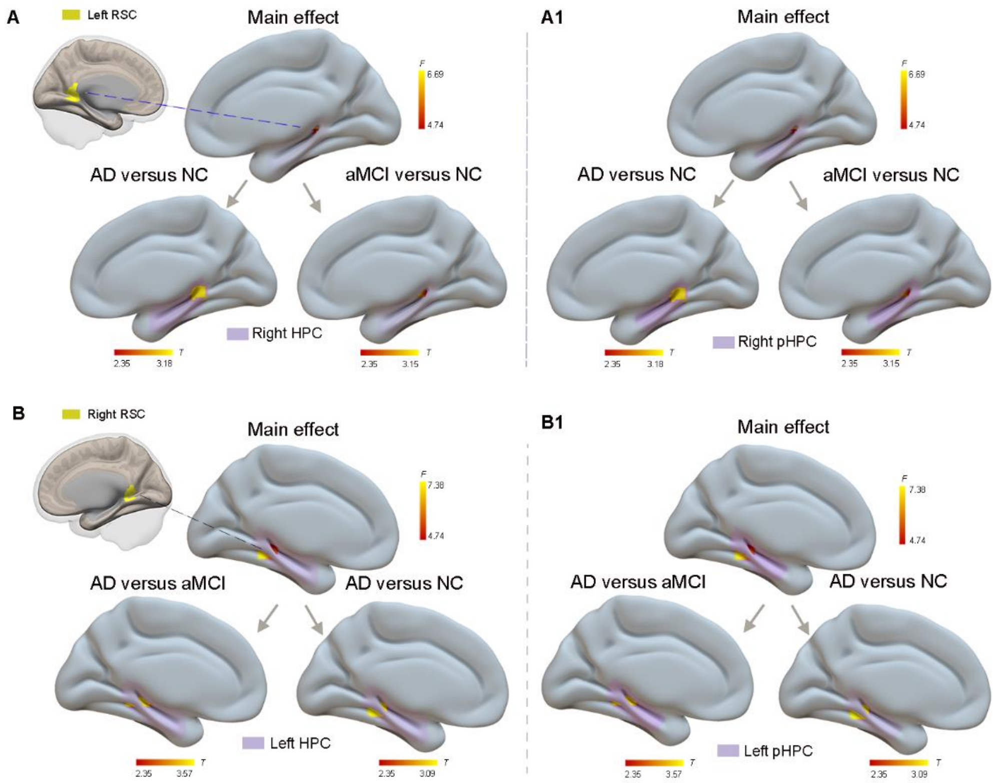

Supplementary Figure 4 Group differences in functional connectivity of the bilateral RSC with the HPC and the hippocampal subregions (with no smoothing, and semipartial correlations) among the AD, aMCI, and NC groups. (A) Regions showing altered functional connectivity between the left RSC and the right HPC among the three groups (mean ± SD are reported from n=162 participants, one-way ANOVAs, with Bonferroni's method for pairwise comparisons). (A1) Regions showing altered functional connectivity between the left RSC and the right posterior HPC among the three groups (mean ± SD are reported from n=162 participants, one-way ANOVAs, with Bonferroni's method for pairwise comparisons). (B) Regions showing altered functional connectivity between the right RSC and the left HPC among the three groups (mean ± SD are reported from n=162 participants, one-way ANOVAs, with Bonferroni's method for pairwise comparisons). (B1) Regions showing altered functional connectivity between the right RSC and the left posterior HPC among the three groups (mean ± SD are reported from n=162 participants, one-way ANOVAs, with Bonferroni's method for pairwise comparisons). RSC, retrosplenial cortex; HPC, hippocampus; pHPC, posterior hippocampus; AD, Alzheimer's disease; aMCI, amnestic mild cognitive impairment; NC, normal controls.

Supplementary Table 2 Comparison of functional connectivity with no smoothing, and semipartial correlations among the HC, aMCI, and AD groups

Comparison	Brain regions	Side	Cluster size	*F/T* value	Peak MNI coordinates		
					X	Y	Z
Left RSC							
Main effect	HPC	R	14	6.69	30	-40	0
AD < NC	HPC	R	47	3.18	30	-40	0
aMCI < NC	HPC	R	21	3.15	30	-40	0
Main effect	pHPC	R	14	6.69	30	-40	0
AD < NC	pHPC	R	47	3.18	30	-40	0
aMCI < NC	pHPC	R	21	3.15	30	-40	0
Right RSC							
Main effect	HPC	L	21	7.38	-32	-40	-6
AD < NC	HPC	L	37	3.57	-34	-38	-10
AD < aMCI	HPC	L	21	3.09	-32	-36	-6
Main effect	pHPC	L	21	7.38	-32	-40	-6
AD < NC	pHPC	L	37	3.57	-34	-38	-10
AD < aMCI	pHPC	L	21	3.09	-32	-36	-6

Note: Brain regions showed significant differences in functional connectivity among the three groups (Gaussian random field (GRF) corrected (voxel $p < 0.01$, cluster $p < 0.05$)).

Abbreviations: RSC, retrosplenial cortex; HPC, hippocampus; pHPC, posterior hippocampus; L, left; R, right; MNI, Montreal neurological institute; NC, normal controls; aMCI, amnestic mild cognitive impairment; AD, Alzheimer's disease.

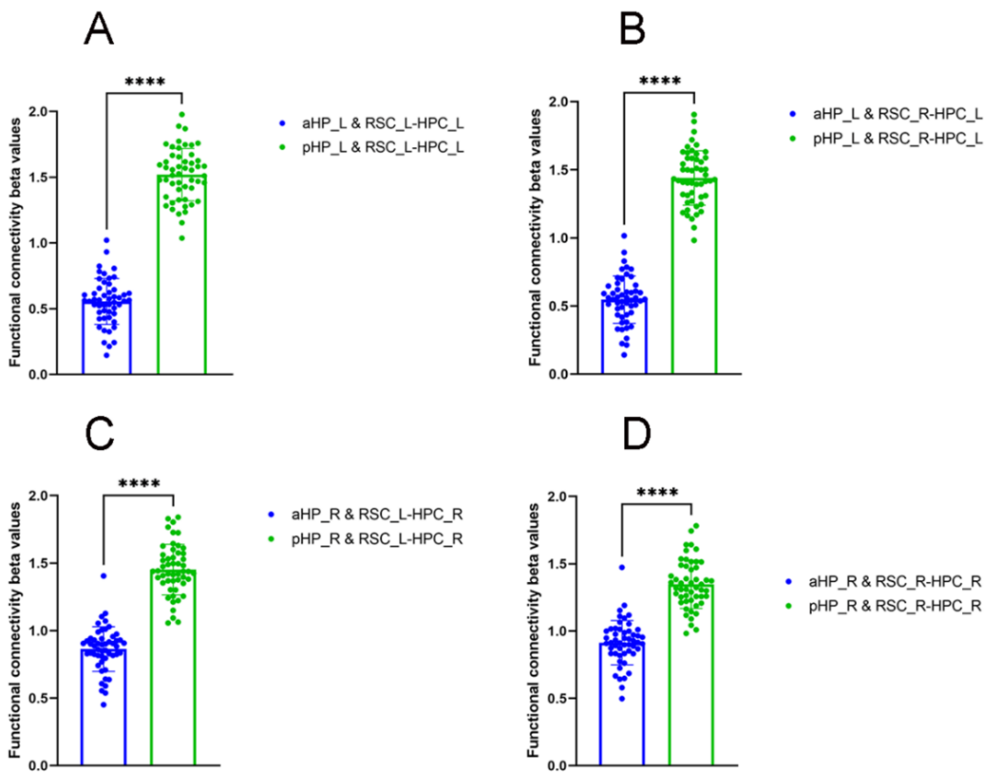

Supplementary Figure 5 Comparison of the anterior HPC and posterior HPC functional connectivity with the **areas which had significant connectivity with the RSC.** (A-D) Functional connectivity between the significant connectivity results and the ROI boundaries of posterior HPC was significantly higher than that of connectivity with the ROI boundaries of anterior HPC (using the paired samples t-test in the NC group (n = 51)). Bar plots displayed mean functional connectivity beta values. (A) Blue dots and green dots represent mean functional connectivity values of left aHP or left pHP with the significant connectivity results between the left RSC and left HPC, respectively. (B) Blue dots and green dots represent mean functional connectivity values of left aHP or left pHP with the significant connectivity results between the right RSC and left HPC, respectively. (C) Blue dots and green dots represent mean functional connectivity values of right aHP or right pHP with the significant connectivity results between the left RSC and right HPC, respectively. (D) Blue dots and green dots represent mean functional connectivity values of right aHP or right pHP with the significant connectivity results between the right RSC and right HPC, respectively. **** Statistically significant at the 0.0001 level. RSC, retrosplenial cortex; HPC, hippocampus; pHP, posterior hippocampus; aHP, anterior hippocampus; L, left; R, right.

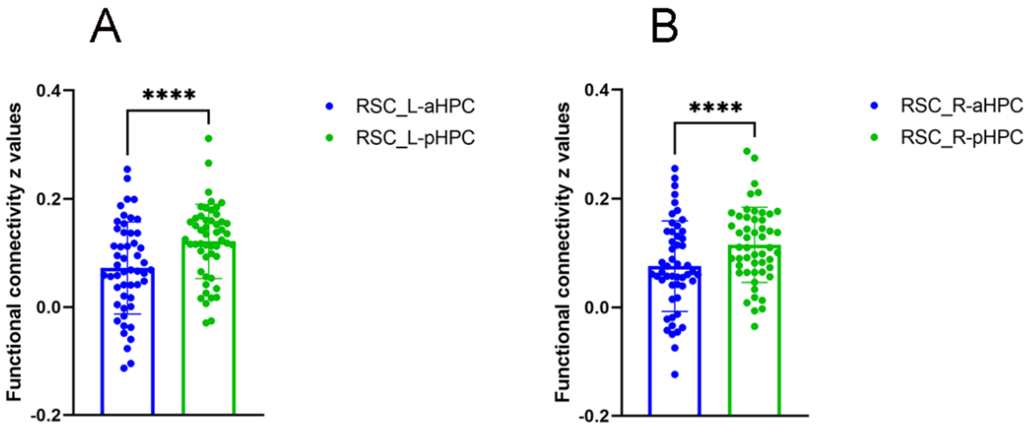

Supplementary Figure 6 Comparison of the **anterior HPC and posterior HPC functional connectivity with** the RSC. (A) The difference of functional connectivity between the RSC_L with aHPC and the RSC_L with pHPC (using the paired samples t-test in the NC group (n = 51)). (B) The difference of functional connectivity between the RSC_R with aHPC and the RSC_R with pHPC (using the paired samples t-test in the NC group (n = 51)). Bar plots displayed mean functional connectivity z values for the NC group. (A) Blue dots represent mean functional connectivity values of left RSC with the aHPC and green dots represent mean functional connectivity values of left RSC with the pHPC. (B) Blue dots represent mean functional connectivity values of right RSC with the aHPC and green dots represent mean functional connectivity values of right RSC with the pHPC. ****Statistically significant at the 0.0001 level. RSC, retrosplenial cortex; HPC, hippocampus; pHPC, posterior hippocampus; aHPC, anterior hippocampus; L, left; R, right.
